# Supplementary material for: Resolving Dirac electrons with broadband high-resolution NMR
Source: Nat Commun. 2020 Mar 9;11:1285. doi: 10.1038/s41467-020-14838-4 (PMC7062727; doi:10.1038/s41467-020-14838-4)
Supplement: Supplementary file 1 — Supplementary Information [file 41467_2020_14838_MOESM1_ESM.pdf]

SUPPLEMENTARY INFORMATION FOR

"Resolving Dirac Electrons with broadband high resolution NMR"

Wassilios Papawassiliou[1], Aleksander Jaworski[1], Andrew J. Pell[1]*, Jae Hyuck Jang [2], Yeonho Kim[2], Sang-Chul Lee[2], Hae Jin Kim[2]*, Yasser Alwahedi[3, 4], Saeed Alhassan[3], Ahmed Subrati[3, 5], Michael Fardis[6], Marina Karagianni[6], Nikolaos Panopoulos[6], Janez Dolinsek[7], and Georgios Papavassiliou[6]*.

[1] Department of Materials and Environmental Chemistry, Arrhenius Laboratory, Stockholm University, Svante Arrhenius vag 16 C, SE-106 91 Stockholm, Sweden,

[2] Electron Microscopy Research Center, Korea Basic Science Institute, 169-148 Gwahak-ro, Yuseong-gu, Daejeon 34133, Republic of Korea,

[3] Department of Chemical Engineering, Khalifa University, PO Box 2533, Abu Dhabi, United Arab Emirates,

[4] Center for Catalysis and Separation, Khalifa University of Science and Technology, P.O.Box 127788, Abu Dhabi, UAE,

[5] NanoBioMedical Centre, Adam Mickiewicz University, Wszechnicy Piastowskiej 3, 61-614 Poznań, Poland,

[6] Institute of Nanoscience and Nanotechnology, National Center for Scientific Research "Demokritos", 153 10 Aghia Paraskevi, Attiki, Greece,

[7] J. Stefan Institute and University of Ljubljana, Faculty of Mathematics and Physics, Jamova 39, SI-1000 Ljubljana, Slovenia

*andrew.pell@mmk.su.se, hansol@re.kbsi.kr, g.papavassiliou@inn.demokritos.gr.

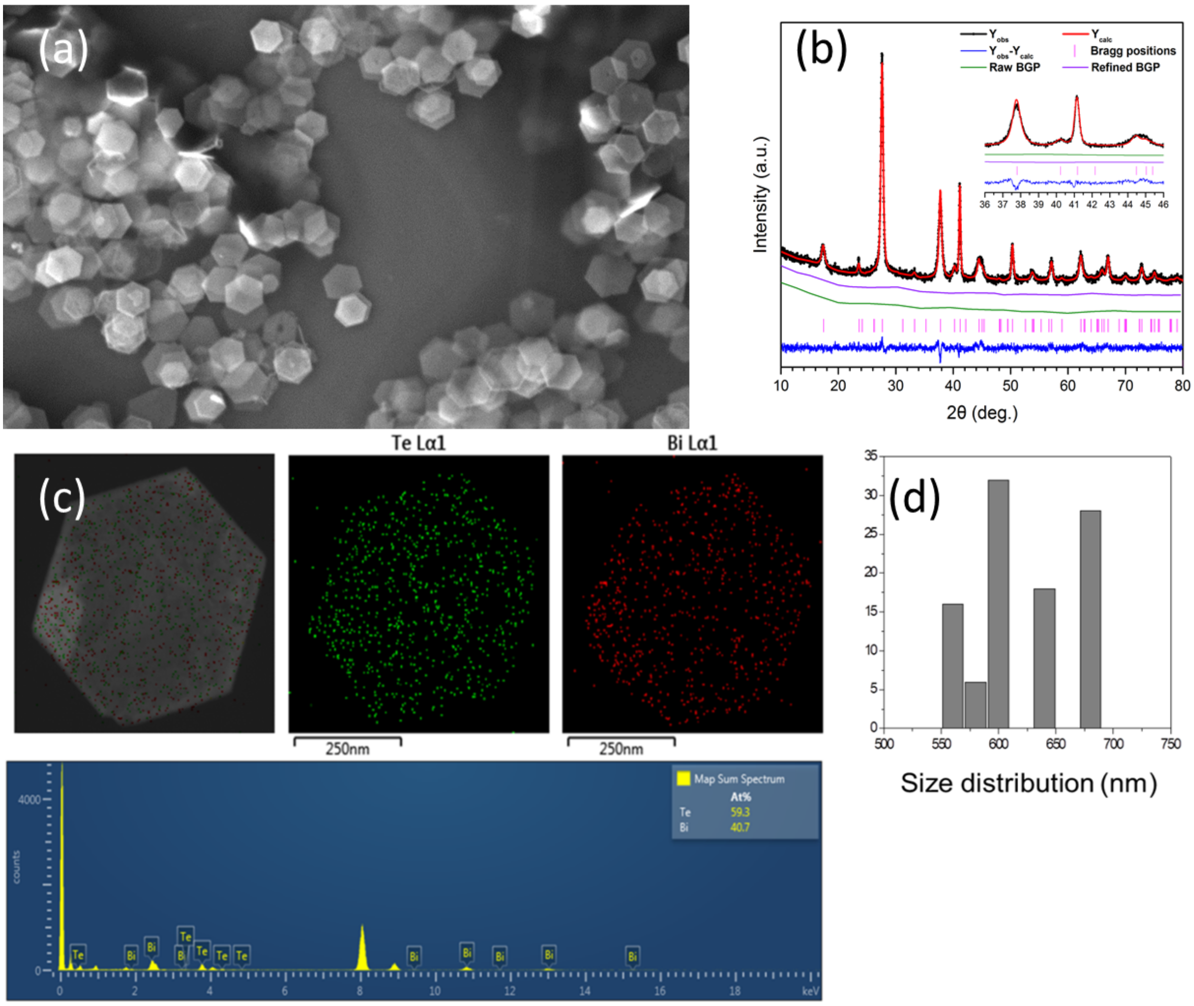

Supplementary Figure 1: Scanning Electron Microscopy (SEM) of Bi_2Te_3 nanoplatelets. (a) Low-Magnification SEM image. (b) The experimental XRD pattern (black line) and the Rietveld analysis (red line) of the Bi_2Te_3 nanoplatelets. (c) Dark-field SEM with Te and Bi atomic mapping and EDX results with inset showing the atomic content percentage of Bi and Te. (d) Size distribution of the Bi_2Te_3 nanoplatelets.

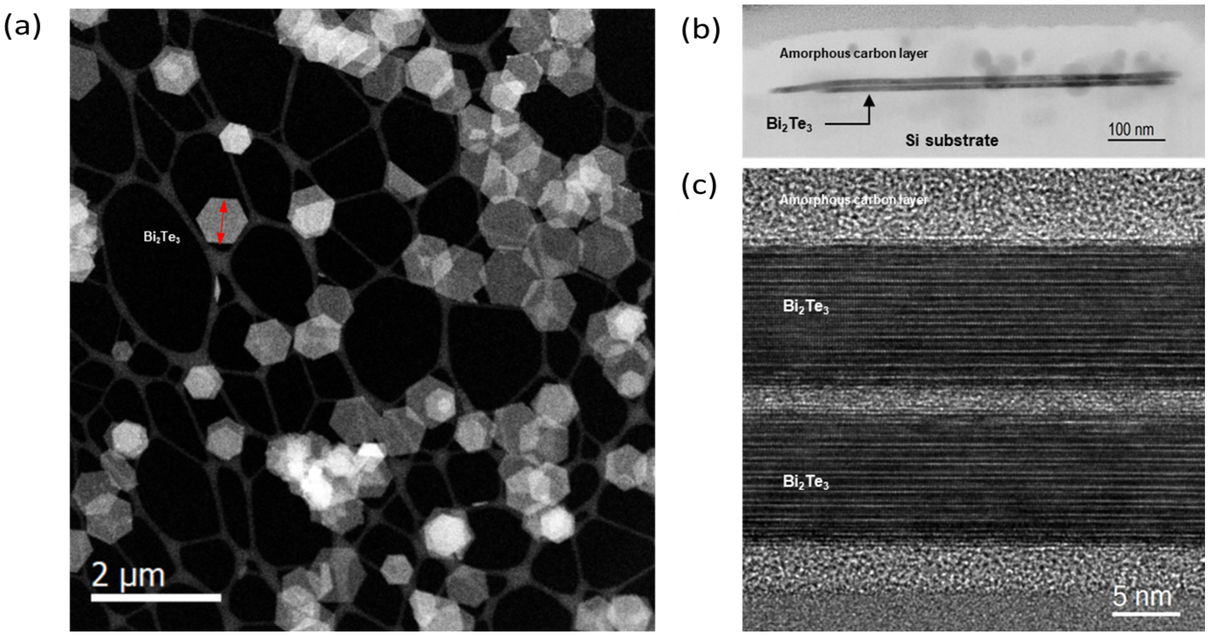

Supplementary Figure 2: Transmission Electron Microscope micrographs of Bi_2Te_3 nanoplatelets. (a) The low-magnification HAADF image shows the overall uniform size and hexagonal shape of the Bi_2Te_3 nanoplatelets. (b) A low-magnification TEM image in which the cross-section of two Bi_2Te_3 nanoplatelets is observed. (c) The magnified TEM image of the two Bi_2Te_3 nanoplatelets in Figure S2b. The two Bi_2Te_3 nanoplatelets are separated by an amorphous layer (grey), and have thicknesses 10.4 nm and 9.5 nm, respectively.

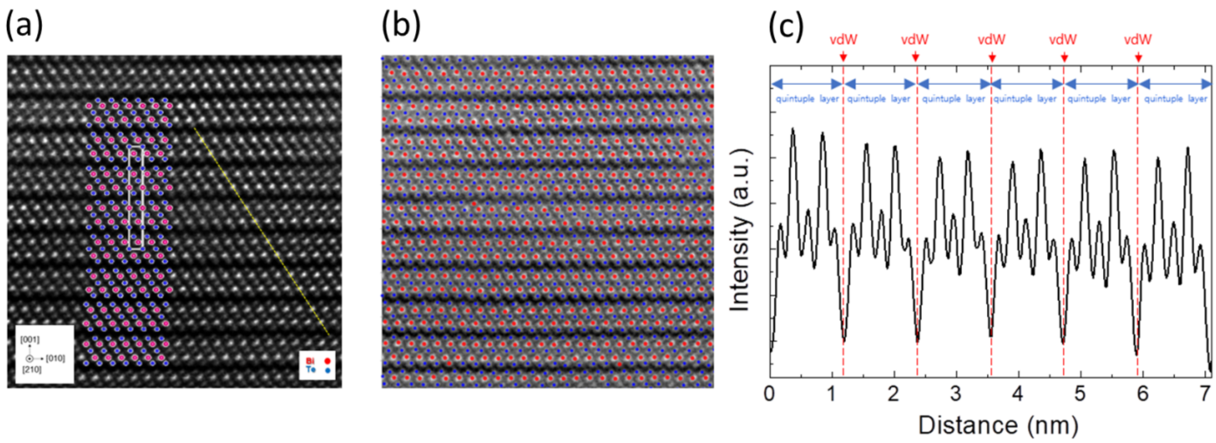

Supplementary Figure 3: Atomic position mapping of Bi_2Te_3 and intensity profiles of atomic columns. (a) High-resolution cross-sectional HAADF image of Bi_2Te_3 along the [210] direction. The red and blue dots represent the Bi and Te columns, and the atomic model of the Bi_2Te_3 [210] direction overlays the HAADF image. (b) Chemical Analysis Image of the Bi_2Te_3 nanoplatelet. (c) Intensity profile along the yellow line in panel (a) shows the separation of the Bi and Te columns in quintuple layers. Van der Waals bonding is indicated between the quintuple layers.

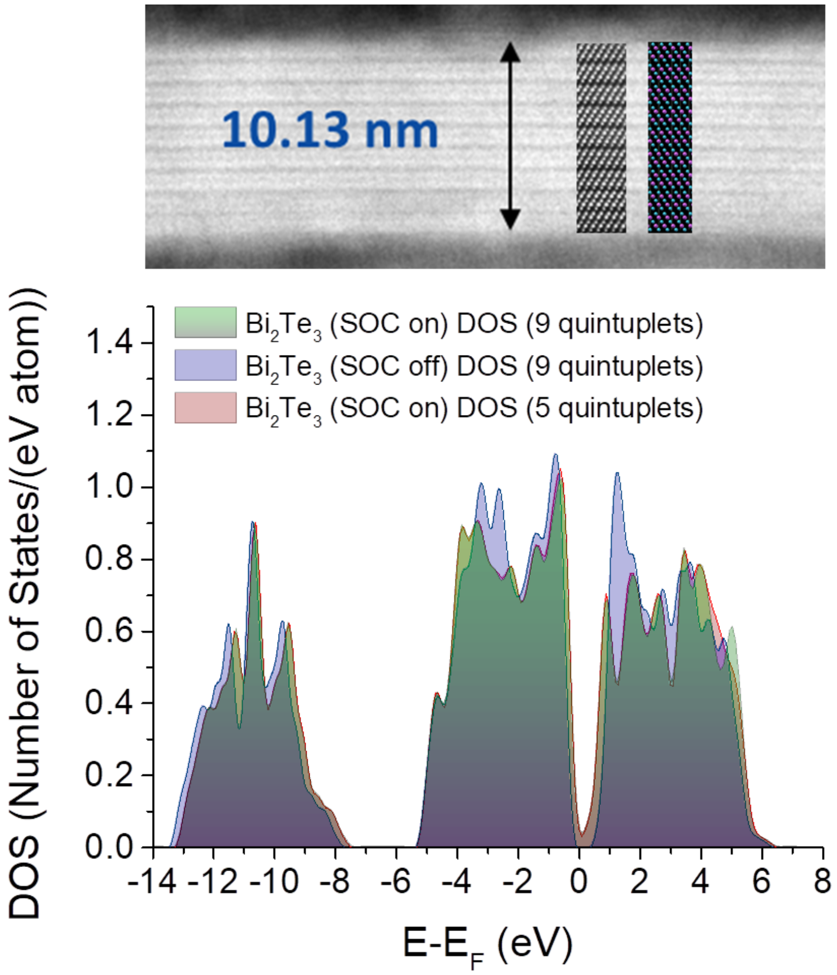

Supplementary Figure 4: DOS of Bi_2Te_3 slabs comprising both 5 quintuplets and 9 quintuplets calculated with and without SOC. The 9-quintuplets slab corresponds to the average nanoplatelet thickness as seen in the upper panel of the Figure. When SOC is switched off an energy gap of ~200 meV opens at the Fermi level between the highest Valence Band and the Lowest Conduction Band. The extra DOS that is present in this gap when SOC is included is due to the surface Dirac states.

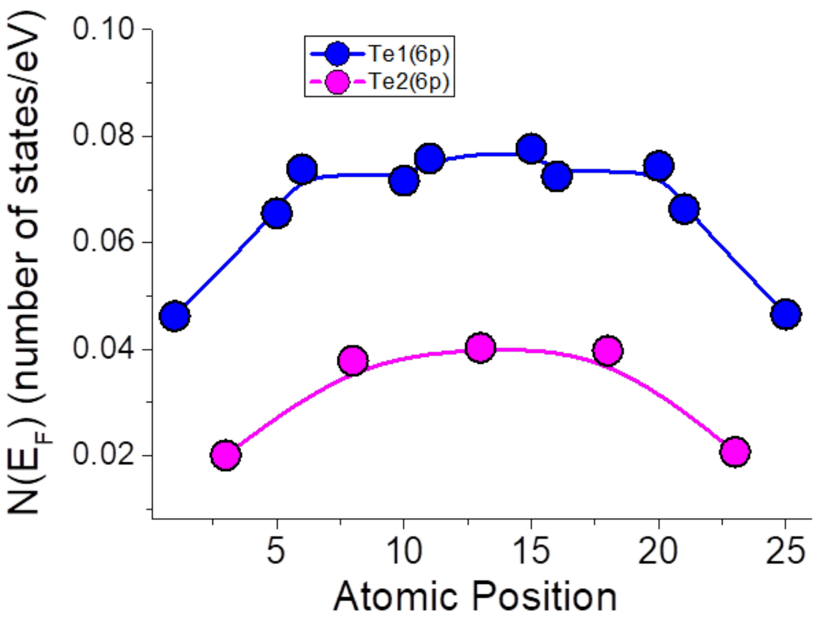

Supplementary Figure 5: The Te(1) and Te(2) DOS at the Fermi level $N(E_F)$, across a 5-quintuplet (~5 nm) Bi_2Te_3 slab.

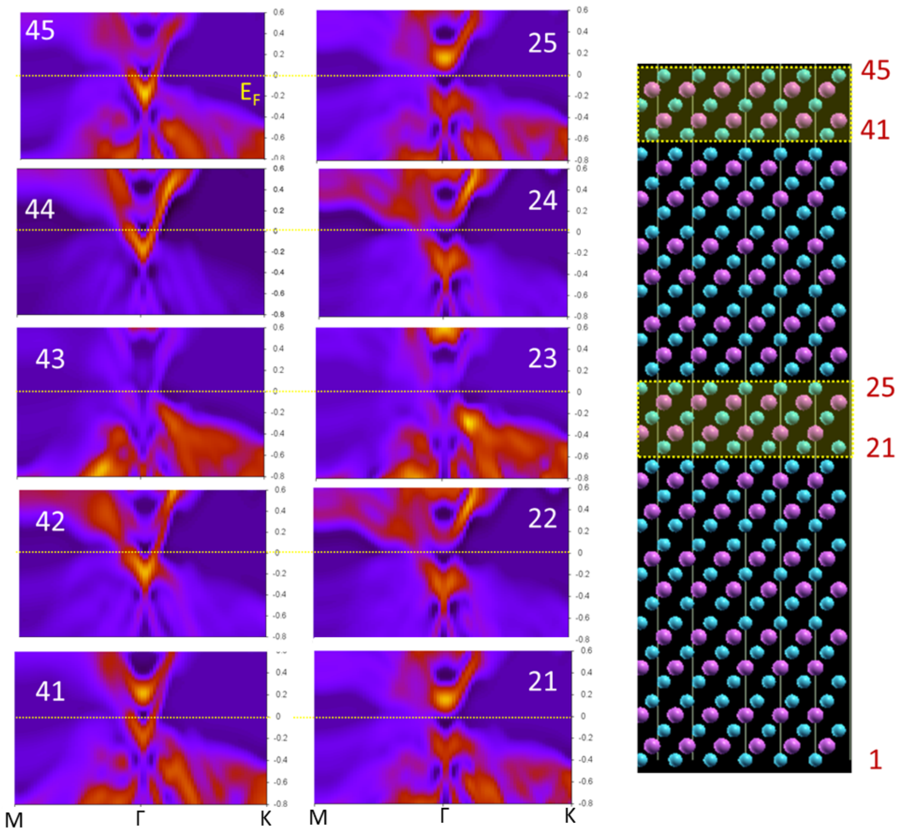

Supplementary Figure 6: *k*-resolved projected DOS in two different *k*-directions of a 9-quintuplets Bi_2Te_3 slab. The cyan spheres in the right-hand image represent Te atoms, and the magenta spheres are Bi atoms. The atomic rows within the slab are numbered 1 to 45. The contribution of the central quintuplet (atomic rows 21-25) to the Dirac states is significantly reduced, as also shown in Figure 4b.

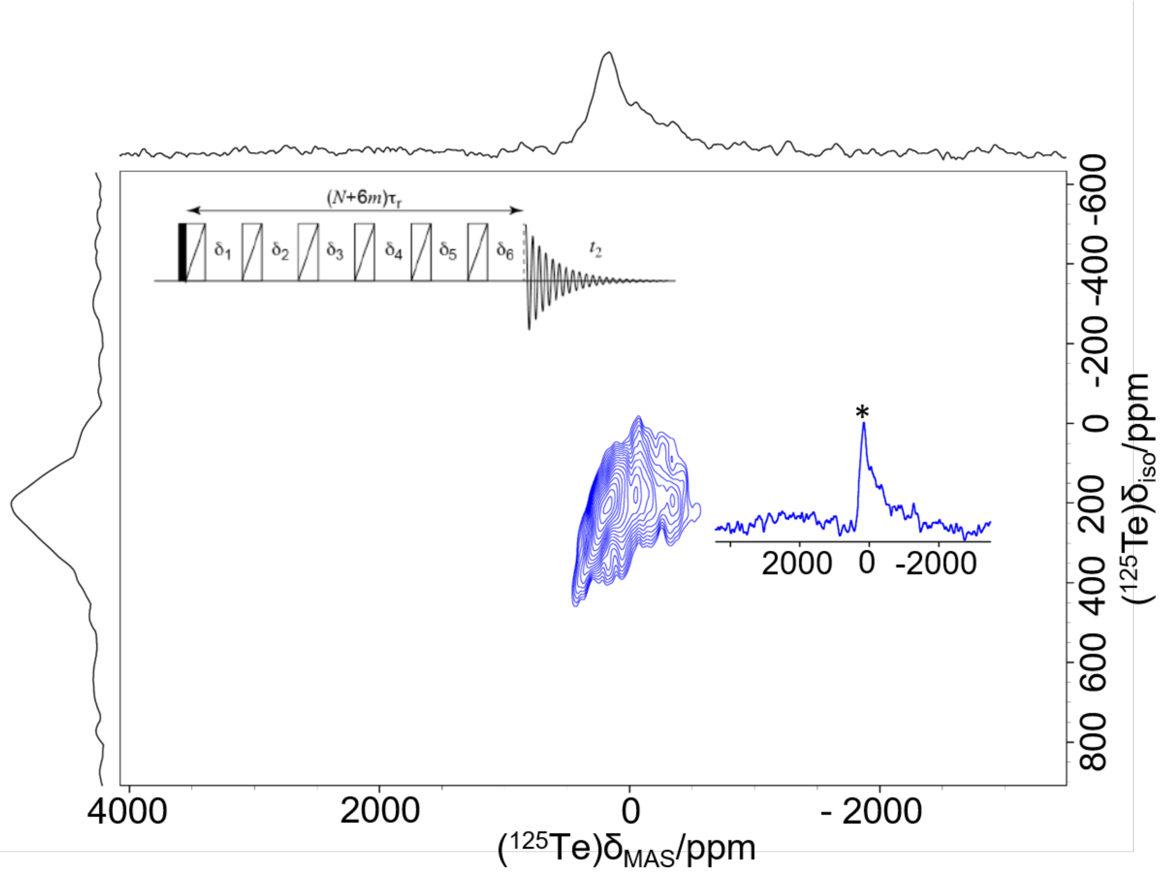

Supplementary Figure 7: ^{125}Te aMAT NMR spectrum of bulk Bi_2Te_3. Separation of the chemical shift and the chemical shift anisotropy is achieved in the bulk material. A single spectral feature, which comprises two overlapping resonances due to the two inequivalent Te sites, is observed at 250 ppm in the isotropic (δ_{iso}) projection. Inset: The aMAT pulse sequence containing a 90° excitation pulse followed by six refocusing SHAPs prior to acquisition.

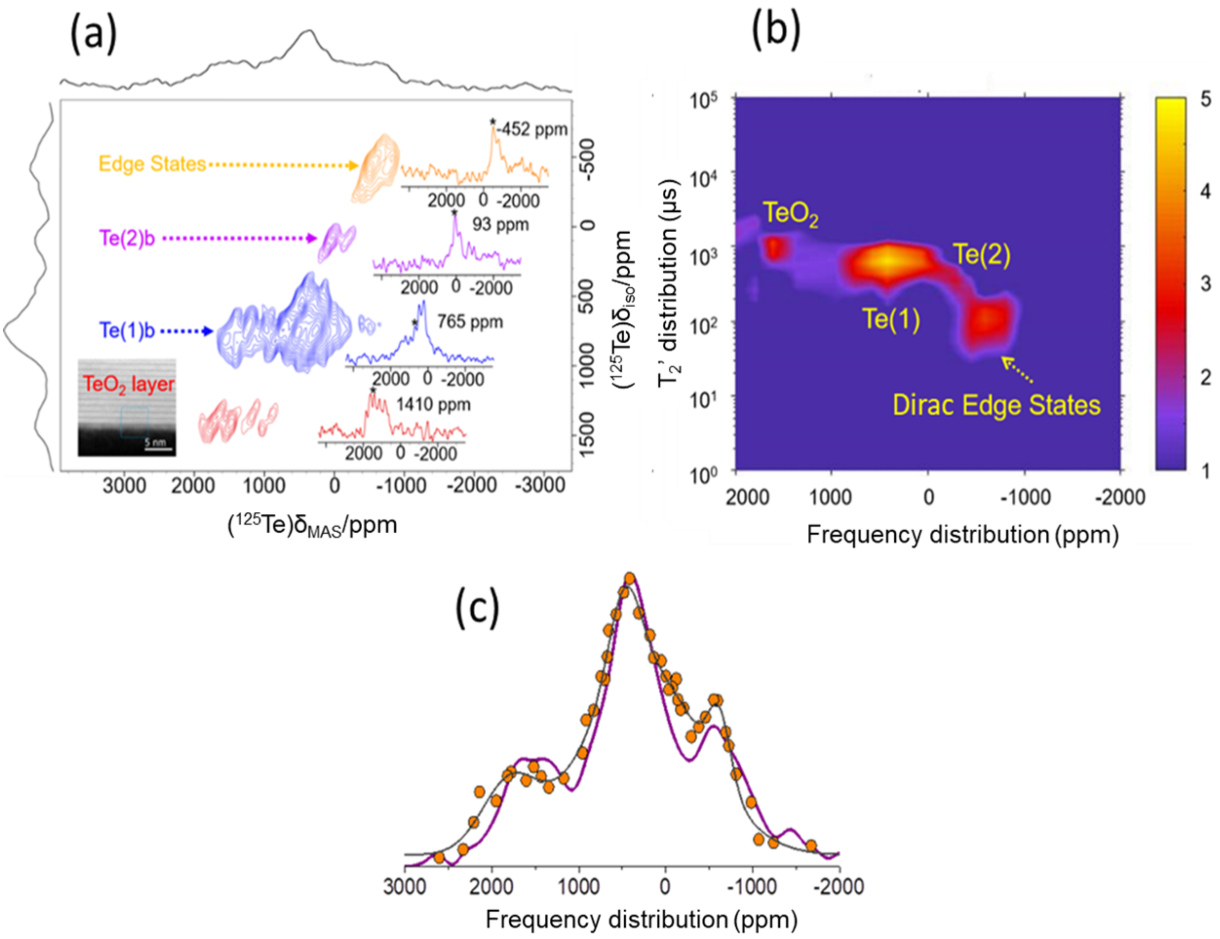

Supplementary Figure 8: ^{125}Te aMAT NMR, T_2' distribution and Double Adiabatic Echo (DAE) of lightly-oxidized Bi_2Te_3 nanoplatelets. (a) Fully relaxed aMAT spectrum, showcasing both the bulk and the edge states of the nanoplatelets, but also exposing a thin TeO_2 layer at 1410 ppm forming at the surface of a few nanoplatelets. (b) Fully-relaxed $\boldsymbol{T_2'}$ distribution spectrum showcasing the same features. (c) The Double Adiabatic Echo (magenta color line) and the frequency sweep spectra (orange circles and black line).

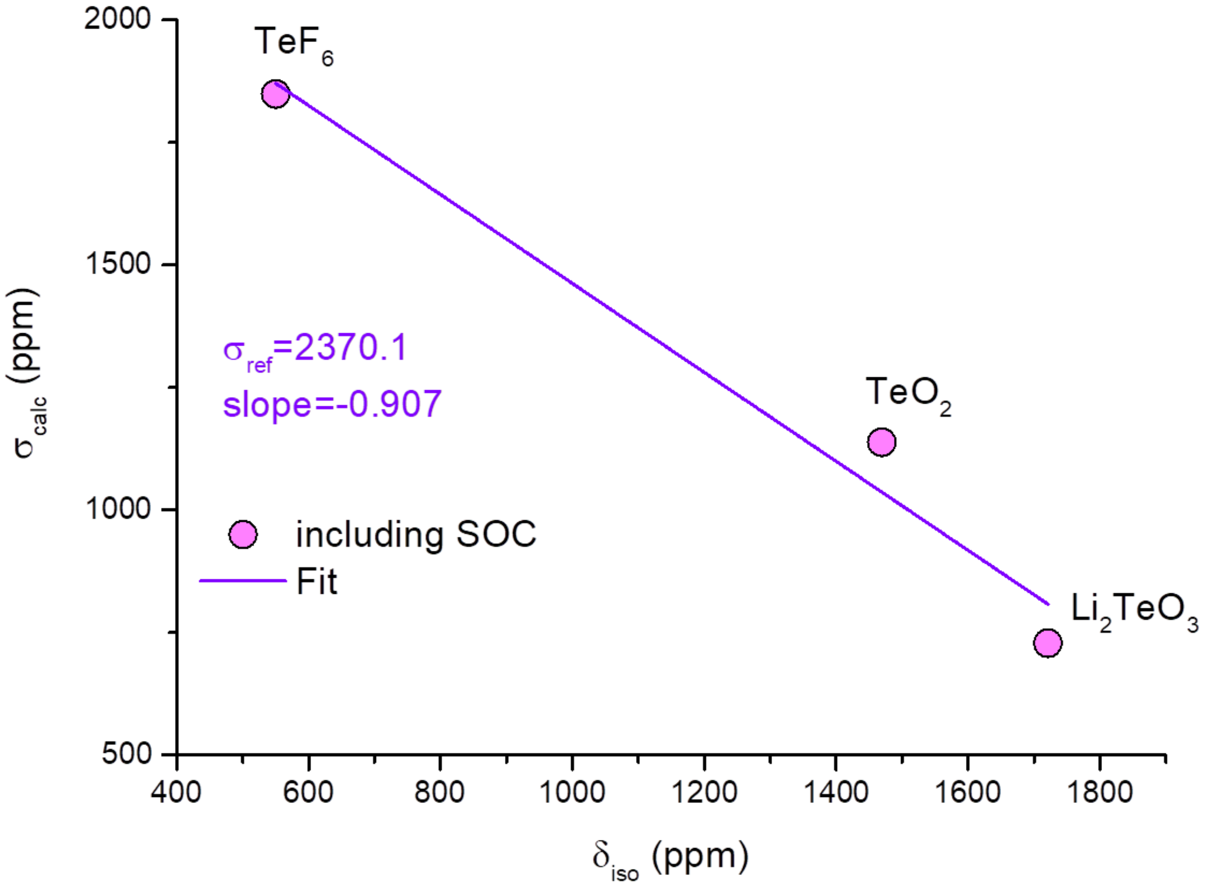

Supplementary Figure 9: Correlation between calculated ^{125}Te NMR isotropic shielding and experimental isotropic chemical shift. Data were considered for three representative compounds. According to the linear fit in the presence of SOC, the reference shielding is equal to $\sigma_{ref} = 2370.1$ ppm, and the slope is -0.907.

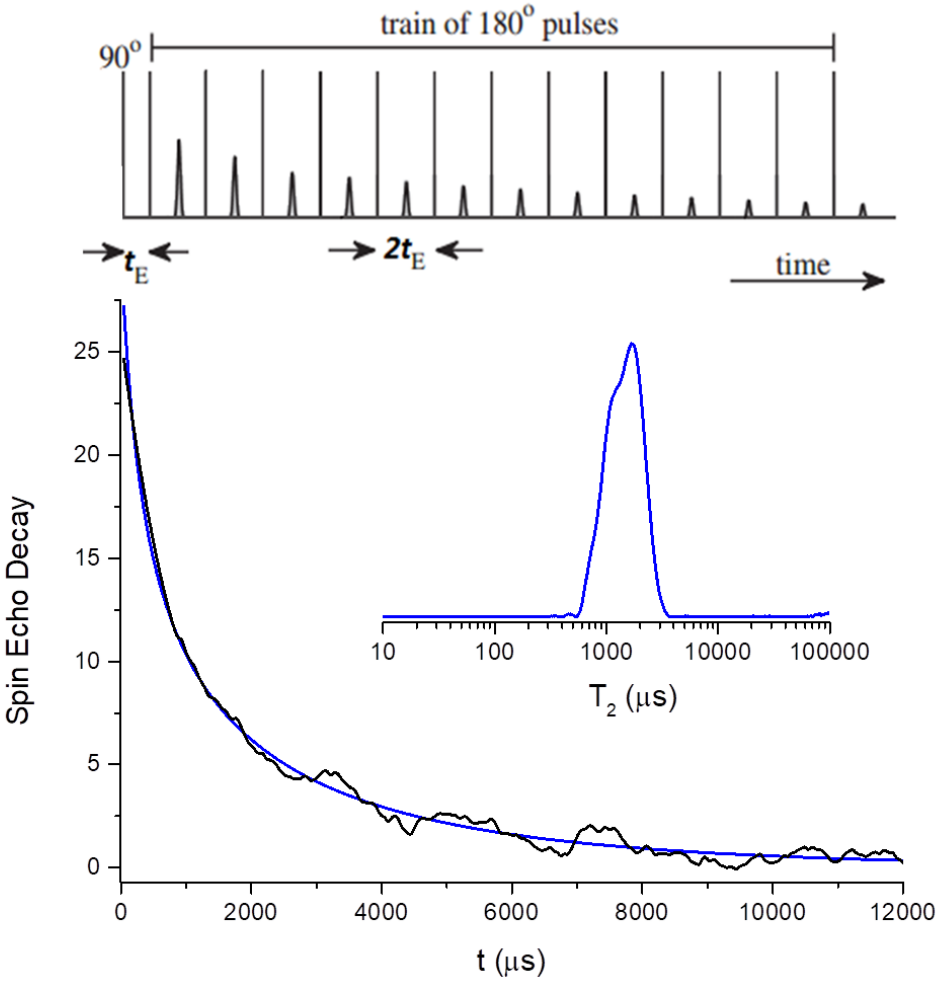

Supplementary Figure 10: The ^{125}Te NMR T_2' distribution (inset) obtained by inverting the experimental spin-echo decay (black curve in the main panel). The experimental echo-train comprises 300 echoes with an interpulse separation of $\mathbf{2}\boldsymbol{t}_{\mathbf{E}} = \mathbf{40}$ μs. The inversion was performed by implementing a non-negative Tikhonov regularization algorithm. The blue curve in the main panel is the best monoexponential fit to the experimental spin-echo decay.

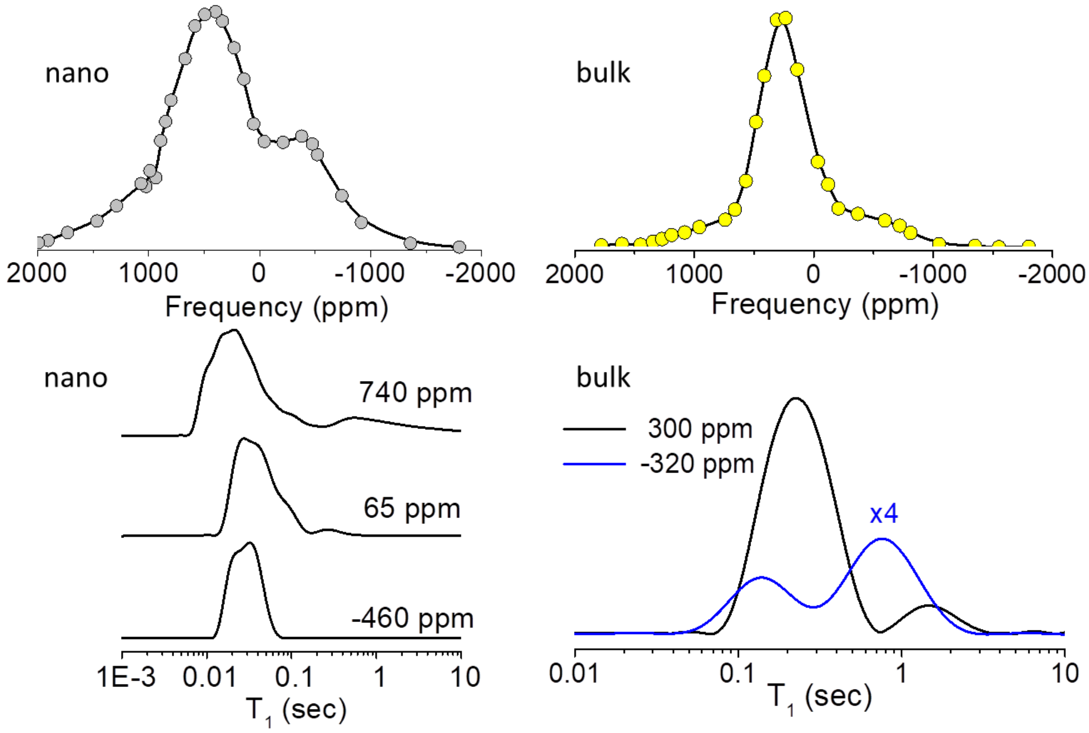

Supplementary Figure 11: The ^{125}Te NMR spin-lattice relaxation time T_1 distribution of the nanoplatelets (left panels) and the bulk system (right panels), at characteristic frequencies. For clarity, the signal intensities have been rescaled. In the case of the nanoplatelets, T_1s were measured at frequencies close to the three isotropic peaks observed in the aMAT spectrum. The T_1 values of the nanoplatelets are an order of magnitude shorter, reflecting the significant role of the Dirac electrons on the NMR relaxation processes across the entire nanoplatelet.

Supplementary Note 1: Electron Microscopy (SEM/TEM) and XRD morphological and compositional analysis of the Bi_2Te_3 nanoplatelets

Supplementary Figure 1 presents the morphological characteristics of pristine Bi_2Te_3 nanoplatelets. Hexagonal (0001) facets can be seen in Supplementary Figure 1a. The Rietveld refinement presented in Supplementary Figure 1b clearly shows a plausible match with the employed theoretical model with no interference from the selected background (BGP). The diffraction pattern can be indexed to JCPDS: 15-0863. The inset shows the high quality and credibility of the refinement. The stoichiometry maps in Supplementary Figure 1c show homogeneous atomic distribution of constituents with stoichiometric higher Te intensity. The EDX results show Bi and Te signals with very good match to the ideal stoichiometry, i.e. 40.7 at.% Bi and 59.3 at.% Te. The size distribution is quite narrow with the majority of nanoplatelets sized around 600 nm (Supplementary Figure 1d).

Supplementary Figure 2a shows a low magnification High-Angle Annular Dark-Field (HAADF) image, whilst Supplementary Figures 2(b,c) show a cross-sectional TEM image of two nanoplatelets. The average size of the fabricated nanoplatelets is ~10 nm. The High resolution cross-sectional HAADF image of Bi_2Te_3 along the [210] direction in Supplementary Figure 3 shows the excellent stoichiometry and crystal structure of the synthesized samples. It is furthermore noticed that the excellent Rietveld analysis of the XRD pattern and the X-ray energy dispersion spectroscopy results presented in Supplementary Figure 1 confirm the absence of any Te-based impurity phase.

Supplementary Note 2: DFT calculations on the Density of States (DOS) and k-resolved band structure analysis of the Bi_2Te_3 nanoplatelets.

The DOS of a 5-quintuplets Bi_2Te_3 slab (25 atoms in the supercell) and of a 9-quintuplet Bi_2Te_3 slab (45 atoms in the supercell) were calculated in order to examine the role of the various atomic orbitals to the Dirac electron states and subsequently to the ^{125}Te NMR Knight shifts. Each quintuplet comprises of a Te(1)-Bi-Te(2)-Bi-Te(1) atomic arrangement, whereas successive quintuplets are bonded to each other with van der Waals forces.

Supplementary Figure 4 shows the total DOS of both Bi_2Te_3 slabs with and without Spin Orbit Coupling (SOC). In the absence of SOC, a 200 meV energy gap opens between the Valence Band Maximum (VBM) and the Conduction Band Minimum (CBM). When SOC is turned on, the gap is closing and a finite DOS is crossing the Fermi level, which looks to be

almost the same in both atomic arrangements. In the case of slabs thinner than 5 quintuplets an energy gap was observed to open at the Dirac point.

Supplementary Figure 5 shows Te(1) and Te(2) DOS at the Fermi level $N(E_F)$, across a 5-quintuplet (~5 nm) Bi_2Te_3 slab. Dirac electrons are seen to extend uniformly across the nanoplatelets.

Supplementary Figure 6 presents the calculated k-resolved projected DOS of the outermost and central quintuplets of the 45 atoms Bi_2Te_3 slab. It is clearly seen that atoms in the outermost quintuplets have sufficiently higher contribution to the Dirac electron DOS. Evidently, by increasing the slab thickness the projected DOS of atoms in the central quintuplets attains a “bulk-like” character.

Supplementary Note 3: ^{125}Te aMAT NMR of microcrystalline Bi_2Te_3 and slightly oxidized Bi_2Te_3 nanoplatelets.

To determine the isotropic Knight shift of the two non-equivalent Te sites the aMAT pulse sequence was employed. This is a constant period pulse train of six short high-power adiabatic pulses (SHAPs) following an initial 90° excitation pulse, as shown in the inset of the Supplementary Figure 7. The main panel of the Supplementary Figure 7 highlights the strength of the aMAT experiment, which is able to eliminate anisotropies, unveiling the presence of just a single broad Knight shift containing the two overlapping, non-equivalent Te sites.

Supplementary Figure 8 showcases the excellent resolution that can be achieved when performing the aMAT experiment on the ultrathin Bi_2Te_3 nanoplatelets. Four well-resolved environments are observed, of which three are attributed to the non-equivalent tellurium sites and the edge states, respectively of Bi_2Te_3, while the fourth is attributed to a small number of surface oxidized nanoplatelets (peak at 1410 ppm). It is noticed that slight oxidization is not influencing Dirac edge states.

Supplementary Note 4: Correlation between experimental isotropic ^{125}Te NMR chemical shift and the DFT-calculated isotropic magnetic shielding.

In order to obtain the reference isotropic ^{125}Te magnetic shielding σ_{ref}, NMR calculations were performed on three prototype materials in the presence of spin-orbit coupling. Crystallographic data for these materials were acquired from https://materialsproject.org to perform the NMR DFT calculations with Wien2k. The data in Supplementary Figure 9 are plots of the calculated isotropic magnetic shielding as function of the experimental isotropic chemical shifts; the latter were taken from refs. (1, 2) and are referenced relative to $(CH_3)_2Te$, for which $\delta = 0$ ppm. The magenta line in Supplementary Figure 9 was fitted to the plot according to formula $\sigma_{calc} = 2370.1 - 0.907\delta_{iso}$. The calculated reference shielding σ_{ref}, defined as the intercept of the best-fit line with the vertical axis, was found to be $\sigma_{ref} = 2370.1$ ppm. To avoid inaccuracies due to temperature dependence of the NMR shifts, which is often relevant for narrow gapped semiconductors, the selected reference materials are insulators with large band gaps.

Supplementary Note 5: Details on the inversion to acquire the ^{125}Te NMR T_2' and T_1 distributions.

The upper panel in Supplementary Figure 10 shows schematically the typical CPMG pulse sequence. Experimental spin-echo decay curves were acquired by recording the intensity of consecutive spin echoes. CPMG spin echoes decay under the effect of the inherent T_2 relaxation, the presence of coherent dephasing, and eventually electron-nuclear interactions coupled with the CPMG pulse train. The overall effective dephasing time constant is defined as T_2'. In case of the Bi_2Te_3 nanoplatelets the presence of spin-diffusion was confirmed by recording the spin echo decay curves at different delay times $2t_E$. The acquired CPMG spin echo decay trains were made of 300 echoes with interecho distance $2t_E = 40$ µs (exemplary experimental spin echo decay is shown as black line in the main panel). The inset shows the $g(T_2')$ distribution after inverting the spin-echo decay curve. The blue line in the main panel is the theoretical fit on the experimental data.

In order to acquire the ^{125}Te NMR spin-spin relaxation time T_2' distribution function $g(T_2')$, the experimental CPMG spin-echo decay curves were modelled with a Fredholm integral equation of the first kind[3,4], $\frac{M(t)}{M(0)} = \int_0^{+\infty} k_0(T_2', t) g(T_2') d(log_{10}T_2')$, where $\frac{M(t)}{M(0)}$ is the normalized CPMG spin echo decay and $k_0(T_2', t) = exp\left(-\frac{t}{T_2'}\right)$. This equation can

be transformed in a vector matrix notation[3] to $\boldsymbol{M} = \boldsymbol{K_0 g}$, which in turn can be inverted to obtain the $g(T_2')$ distribution function. In the present work, the inversion was achieved by implementing a modified non-negative Tikhonov regularization algorithm[4].

The contour plot in Figures 3c and 3d of the main article are made out of 30 $g(T_2')$ curves, acquired at 30 consecutive resonance frequencies, covering the whole ^{125}Te NMR spectra. Soft pulses were used so that each time a narrow frequency bandwidth was irradiated.

The spin-lattice relaxation time distribution function $g(T_1)$ was obtained in a similar way as $g(T_2')$, by replacing the kernel $k_0(T_2', t)$ in the integral equation with $k_0(T_1, t) = 1 - exp\left(-\frac{t}{T_1}\right)$. Supplementary Figure 11 shows the ^{125}Te NMR $g(T_1)$ of the nanoplatelet and bulk systems. In case of the nanoplatelets, T_1's were measured at NMR frequencies very close to the three isotropic resonances of the aMAT spectra. A striking difference in the T_1 values between the bulk and the nanoplatelets systems is observed.

Supplementary References

1. Alkan, F. & Dybowski, C. l. Spin-orbit effects on the ^{125}Te magnetic-shielding tensor: A cluster-based ZORA/DFT investigation. *Solid State Nucl. Magn. Reson.* **95,** 6–11 (2018).
2. Hada, M. et al. Quasirelativistic study of ^{125}Te Nuclear Magnetic Shielding Constants and Chemical Shifts. *J. Comput. Chemistry* **22,** 1502–1508 (2001).
3. Mitchell, J., Chandrasekera, T. C. & Gladden, L. F. Numerical estimation of relaxation and diffusion distributions in two dimensions. *Prog. Nucl. Magn. Reson. Spectroscopy* **62,** 34-50 (2012).
4. Day, I. J. On the inversion of diffusion NMR data: Tikhonov regularization and optimal choice of the regularization parameter. *Journal of Magnetic Resonance* **211,** 178-185 (2011).
